# Retraction: The long non‐coding RNA‐ROR promotes osteosarcoma progression by targeting miR‐206

**DOI:** 10.1111/jcmm.18199

**Published:** 2024-03-04

## Abstract

Dan Fei, Guoqing Sui, Yang Lu, Long Tan, Zhao Dongxu, Kewei Zhang. The long non‐coding RNA‐ROR promotes osteosarcoma progression by targeting miR‐206. *J Cell Mol Med*. 2019; 23: 1865–1872. https://doi.org/10.1111/jcmm.14087.

The above article, published online on 19 December 2018 in Wiley Online Library (wileyonlinelibrary.com), has been retracted by agreement between the authors, the journal Editor‐in‐Chief, Stefan Constantinescu, The Foundation for Cellular and Molecular Medicine and John Wiley and Sons Ltd. The retraction has been agreed following an investigation into concerns raised by a third party, which revealed inappropriate duplications of images between this and other articles that were previously published in a different scientific context. Thus, the editors consider the conclusions of this manuscript substantially compromised. Although the authors requested a retraction, when asked to approve the retraction wording, they remained unresponsive.

Dan Fei, Guoqing Sui, Yang Lu, Long Tan, Zhao Dongxu, Kewei Zhang. The long non‐coding RNA‐ROR promotes osteosarcoma progression by targeting miR‐206. *J Cell Mol Med*. 2019; 23: 1865–1872. https://doi.org/10.1111/jcmm.14087.

The above article, published online on 19 December 2018 in Wiley Online Library (wileyonlinelibrary.com), has been retracted by agreement between the authors, the journal Editor‐in‐Chief, Stefan Constantinescu, The Foundation for Cellular and Molecular Medicine and John Wiley and Sons Ltd. The retraction has been agreed following an investigation into concerns raised by a third party, which revealed inappropriate duplications of images between this and other articles that were previously published in a different scientific context. Thus, the editors consider the conclusions of this manuscript substantially compromised. Although the authors requested a retraction, when asked to approve the retraction wording, they remained unresponsive.

